# Commentary: A Cell Line for Detection of Botulinum Neurotoxin Type B

**DOI:** 10.3389/fphar.2018.01056

**Published:** 2018-09-25

**Authors:** Andy Pickett

**Affiliations:** Toxin Science Limited, Wrexham, United Kingdom

**Keywords:** botulinum toxin, potency assay, mouse assay, alternative assay, commercial issues

The quest for a viable, appropriate and validatable alternative assay to the mouse LD_50_ potency method is something that those working in the field of botulinum toxin (BoNT) have engaged with for decades. The mouse assay has not only been used as the long-standing gold standard for BoNT identification and serotype determination (typically when cases of botulism food poisoning are suspected) but also for the routine assessment and quality release of BoNT commercial product batches used for clinical treatments worldwide.

The literature shows that so-called “alternative assays” have been available since the 1970s (Kozaki et al., 1979; Notermans et al., 1979). However, these early methodologies, together with many of those reported over the intervening 40 years, only detected the presence of the respective BoNT serotype protein, based upon ELISA technology. They detected none of the four key, essential activities of the BoNT molecule—receptor binding, endocytic internalization, Light Chain translocation and enzymatic activity. In the last decade, other modern methodologies have arrived, but these have assessed just one property of the BoNT molecule, typically the enzymatic activity alone (Lindström and Korkeala, 2006). However, this trend is changing: cell-based assays are now available to determine all known properties of the BoNT molecule (Pellett, 2013).

The work of Rust and colleagues, describing an alternative cell-based assay for serotype B BoNT, presents a sound, scientifically determined methodology that could be applied to the single commercial type B product now available (Rust et al., 2017). The authors conclude that “the assay may well be suited for measuring potency of pharmaceutical BoNT/B products and antitoxin antibodies in toxin neutralization testing.” Unfortunately, there are key issues that must be addressed before any such assay is brought into use by a commercial BoNT company. This is also true for similar assays relating to other serotypes, of which there are many.

Rust et al. (2017) are not the first to engineer a cell line for such an application. The US company BioSentinel, Inc. has, for some years, marketed an engineered cell line and accompanying technology as a replacement assay for BoNT potency determinations (Atapattu et al., 2013). The assay system, termed BoCell®, has been automated, making routine quality control use of the method more straightforward (Burlingham et al., 2017). To some extent, the work of Rust and colleagues copies that of BioSentinel: detailed patent searches and evaluations are needed before any exploitation of their method, to determine if there is any infringement of existing patents.

These types of replacement assays need to be rigorously developed and validated before they could be considered acceptable for use by a commercial BoNT company (International Conference on Harmonisation, 2005; Sharma et al., 2008). Also, comparative data with the mouse LD_50_ test must be generated and effective equivalence demonstrated between the methods. The authors have provided none of these data and so an evaluation of the assay in terms of commercial suitability and validity cannot be made. Statements that such an assay could be used commercially therefore fall far short of reality. But there are several other key issues that must be addressed before such a new method can be used.

Firstly, who owns the cell line? Rust and colleagues have engineered the SiMa cell line obtained from the German collection Deutsche Sammlung von Mikroorganismen und Zellkulturen GmbH (DSMZ). But DSMZ are not the original owners of the line, they are simply a repository. The SiMa cell line was first reported by Marini and colleagues at the University of Tuebingen in Germany in 1999 (Marini et al., 1999). The line was derived in August 1991 from a Caucasian male, 20 months-old, with an adrenal neuroblastoma. This makes him the rightful owner. Has DSMZ obtained appropriate permissions to allow distribution and use of his cell line? Did Rust and colleagues have permission to modify the cell line and engineer as they described? The subject of cell line ownership has been highly controversial in recent years and completely clear permissions, coupled to commercial exploitation agreements, must be obtained by any company seeking to use these lines (Greely and Cho, 2013). In addition, the DSMZ ordering terms state clearly “Cell lines and their products shall not be sold or used for commercial purposes or utilized in any other type of commercial activity”: this represents a major problem for a company seeking to use the line. Therefore, aspects about commercial use of the cell line, even whether the engineered version is legal, are unclear. The authors should clarify the situation.

Secondly, who owns the antibodies used in the Rust et al. work? The authors raised a specific, cleaved-VAMP antibody for their assay. Unfortunately, the company used (Davids Biotechnologie Germany) has strict conditions on use of the products they generate: “Unless otherwise specified all products and services from Davids Biotechnologie GmbH are sold for research use only.” A licensing agreement is therefore required (and may not be even possible) if the antibody is to be used for commercial testing of, for example, BoNT type B products. A number of other primary and secondary antibodies have also been used by the group, with suppliers ranging from Synaptic Systems, Abcam and GE Healthcare to “in-house.” This number of companies involved in producing reagents would simply be a negotiation nightmare for a commercial company to deal with, to adopt such an assay. If any of these companies demands a royalty-bearing license agreement, then benefits from having and implementing such an assay diminish rapidly.

The use of antibodies in these alternative assays also raises the issues of provenance of each antibody, together with batch-to-batch reproducibility. The Protein A coated plates from Thermo Fisher Scientific would also fall into this category, due to the biological nature of the Protein A. These data would be essential for submissions to a regulatory authority in any request to substitute the mouse potency assay for a cell-based alternative. The requirements for such data, both historical and newly generated to support the reagent, will lead into deep commercial confidentiality issues with the suppliers. Suppliers can be reticent to supply such data and can demand commercial agreements to, for example, purchase the reagents solely from them, both in specified quantities and over long periods. Such demands negate one of the primary key supply tenants of pharmaceutical product manufacture and testing—second sourcing. Pharma companies strive to have more than one supplier, especially for key raw materials or reagents, to ensure that they are able to continue product manufacture, testing and supply, without pause.

Finally, the NanoLuc® detection system used by Rust and colleagues is a proprietary methodology owned by Promega Corporation, Madison WI, USA. Promega have similar statements, in their terms and conditions of sale, relating to non-commercial use of these products and their NanoLuc® vectors. Licenses for even laboratory research use are required, in some circumstances. The legalities and Limited Use Label Licenses are extensive.

The search for an alternative method to the mouse potency assay has attracted considerable support from many sources over many years, provided by a wide variety of organizations, agencies and government departments. Often, the work has been carried out in an academic department supporting a student(s) studying for higher qualifications: this seems to be the case with the work of Rust and colleagues. Inevitably, the justifications for these projects state that, if the work proposed were to result in a new assay, then this would be available to (or—more strongly—“should be adopted by”) commercial companies. These activities are fruitless (and actually of no value whatsoever) if they are not exploited commercially. In virtually none of these cases—and there are many—have the participants discussed with industry what they are doing and how they propose to develop their approach. They are, essentially, working for themselves alone. The group of Rust and colleagues included no industry representation and no indication that the group had been working with industry, again very disappointing. No matter how sound the science, or how good the results, if the work is designed to be for the benefit of industry then why isn't industry involved?

To date, no alternative BoNT potency assay developed in a university or other institution has been submitted to or accepted by regulatory authorities. Of the two alternatives currently in existence (for Botox® and Xeomin®), both were developed in-house by the manufacturers. The estimated cost of developing the method used for Botox® has been cited as $65 million, an extraordinary amount and well beyond any university programs. Companies who take their own ways forward must be enough testimony to those seeking to develop these alternatives, namely that the commercial world cannot use what they think they can produce. These programs should now cease. The development of alternative assays for BoNT has taken a completely different direction, dealing with the commercial realities that must be taken into account, and no longer needs any input from academia, on any aspect.

## Author contributions

The author confirms being the sole contributor of this work and has approved it for publication.

### Conflict of interest statement

AP is Director and Founder of Toxin Science Limited, Wrexham, UK; Adjunct Professor at the Botulinum Research Center, Institute for Advanced Sciences, North Dartmouth, USA; and was previously Senior Program Leader & Scientific Expert, Neurotoxins, Galderma Uppsala, Sweden. The opinions and statements expressed are those of the author and Toxin Science Limited only.
